# Analysis of the Awareness and Access of Eye Healthcare in Underserved Populations

**DOI:** 10.3390/vision9030055

**Published:** 2025-07-11

**Authors:** Karen Allison, Abdullah Virk, Asma Alamri, Deepkumar Patel

**Affiliations:** 1 Department of Ophthalmology, Flaum Eye Institute, University of Rochester, Rochester, New York, NY 14641, USA; abdullah_virk@urmc.rochester.edu; 2 School of Health Sciences and Practice, New York Medical College, Valhalla, New York, NY 10595, USA; aalamri@student.touro.edu; 3 Prevention of Blindness from Glaucoma and Age Related Macular Degeneration, Floral Park, New York, NY 11001, USA; deepkumarptl@gmail.com

**Keywords:** eye care access, social determinants of health, vision awareness, vision care, eye care

## Abstract

Introduction: Visual impairment impacts millions of people around the world, with the vast majority of problems being treatable. Disadvantaged communities are unable to utilize the same resources to treat these problems due to a lack of knowledge or resources, in addition to the presence of barriers preventing access. The objective of this paper is to assess eye health awareness and evaluate the barriers for individuals from disadvantaged communities in order to inform future interventions and increase access to care. Methods: This is a pilot study utilizing an online anonymous questionnaire designed to assess the demographics, eye health awareness, and access to eye care of community-based patients. A comprehensive literature review was also conducted using PubMed, Scopus, and Google Scholar to evaluate barriers to eye care and methods to improve community health outcomes. The primary goal was to improve understanding of eye health awareness and access in order to inform future strategies that can help in improving eye health awareness and service availability. Results: The results indicated that 61.2% of respondents believed that eye exams are very important, and only 7.7% of participants believed that regular eye exams are not important. The majority of participants (75%) agree that regular eye exams help prevent serious eye conditions and 84.5% believe that eye health can affect quality of life. 35.6% of participants reported they had their eyes checked by a healthcare professional within the last year, while 21.2% reported never having an eye exam. Although the majority of participants found access to eye care services in their community somewhat or very easy, 8.6% and 9.5% of participants found access difficult and very difficult, respectively. Even though 45.6% of participants reported not facing any barriers regarding access to eye care, the cost of services, long waiting times, and lack of nearby eye care providers were often cited as barriers from the remainder of the participants. Moving forward, local interventions such as mobile eye clinics, public health workshops, and telehealth are viable options to obtain an understanding of the community’s health status in addition to creating opportunities to educate and provide health screenings. Conclusion: The results indicate that although there is awareness of the importance of eye health for the majority of participants, there is still a sizable minority who have insufficient understanding. Barriers to healthcare such as cost, waiting times, and proximity to providers are common problems that are preventing many from seeking eye care. Future interventions should be created to increase access and literacy amongst the community through telehealth, mobile eye clinics, and public health workshops. Additional efforts should be taken by healthcare stakeholders to enhance care delivery, implement policies, and improve awareness.

## 1. Introduction

The importance of eye health cannot be overstated, as vision is one of the most fundamental senses. Quality of vision impacts the daily functions of an individual, their quality of life, and also opportunities to learn and make a living. However, in many populations, there is still a massive deficit of access to eye care, combined with very low public education, perception of what is adequate eye care, and why it is so significant. Untreated refractive problems are more common among low-income groups as per the World Health Organization (WHO) report of 2023 [1,2]. Also, near vision impairment is 80% more probable not to be treated among the lower-income regions compared to only 10% in the higher-income regions [1,3]. Moreover, studies have found that a significant portion of visually impaired people are from low and middle-income countries [4]. In the US, many visual problems such as blindness, glaucoma, and diabetic retinopathy are more prevalent in minorities such as African Americans [5,6]. Underserved areas often face major challenges in accessing basic healthcare services, let alone niche services such as eye health. This has not only resulted in eye health being neglected but has also led to an increase in preventable visual impairments. According to WHO, out of 2.2 billion cases of both near and distance vision impairment, at least 1 billion could have been prevented or are yet to be addressed [1]. Due to the rapidly growing populations, especially in regions such as Asia, the total cases of blindness worldwide has only increased over the past three decades, with cataracts being a primary contributor [7,8]. The increase in ocular disorders due to the growing worldwide population is also seen with other ocular disorders such as glaucoma, as it is estimated to affect 111.8 million adults aged 40–80 years old in 2040, particularly impacting Asia and Africa [9].

There is a strong correlation between eye health and educational outcomes. WHO (2023) cites uncorrected refractive error as the leading cause of vision impairment among children everywhere [1]. However, children from underserved areas such as the low-income communities that have lower access to eye health, decreased educational resources about ophthalmic screenings, and fewer funds for eye exams and spectacles suffer the greatest impact. Any uncorrected refractive errors in pediatric populations can result in long-term visual impacts with increased risk for conditions like amblyopia which cause long-term reduced vision in one eye or both eyes [10]. The undetected vision issues among these children ultimately hinder a child’s ability to learn and participate in the education process effectively. Moreover, according Tegegn et al., low levels of education were a key risk factor in eye health awareness [11]. People with eye defects and without a formal education were 97.9% likely to develop blindness as compared to those with a formal education (2% likelihood of developing blindness) [11]. However, underserved areas are the most likely to have poor education facilities or none at all, leading to higher incidences of illiteracy. This, in turn, lowers the community’s health awareness, resulting in higher incidences of unaddressed health issues including vision and ocular diseases, further creating increased barriers for disadvantaged communities.

Lack of treatment for visual impairment in adults may limit employment opportunities along with many other effects on other aspects in their life. Williams & Sahel found that visual impairment was associated with being unable to work [12]. Consequently, the respondents in the study who were employed expressed a lower risk of self-reported visual difficulty than those who were unemployed or looking for work. Tegegn et al. also support this assessment in their findings that the risk of visual impairment was 1.5 times higher for unemployed participants as compared to employed participants [11]. Furthermore, the undiagnosed ocular issues in children resulted in a harder time achieving success in school. This difficulty in education achievement leads to challenges in finding adequate employment and opportunities in higher education, resulting in an endless cycle where people from disadvantaged backgrounds have worse health, making it harder to create wealth for individuals and communities.

The most compelling reason to study the lack of access to and awareness of eye care is the preventable nature of many eye conditions. At least 90% of those affected by visual impairment have a preventable or treatable causes with highly cost-effective interventions [13]. By the year 2050, there might be more than 895 million people with distance vision impairment with 61 million being completely blind, mostly as a result of the preventable causes aforementioned [13]. This means that understanding the barriers to access and awareness is a pivotal point in designing effective preventive strategies. Studies on eye health among underserved areas identify low income, low levels of education, and unemployment, as some of the key factors resulting in the lack of access to eye care services and awareness of eye health. Several researchers have also shown that economic-related factors also pose a significant threat to access to eye care services especially in the low-income populations. A study found that financial barriers affect health amongst Native Hawaiian and Pacific Islander adults; monetary insecurity is associated with eye care avoidance as well as a decline in health status [14]. Economically vulnerable populations cannot afford adequate and timely eye care due to financial reasons, resulting in increased risk of missed appointments and avoidance of receiving care and screening [14,15]. Such populations mostly inhabit places that have few healthcare facilities, resulting in difficulty in even seeking care. In these situations, geographical isolation not only results in delayed presentation and treatment but can also adversely affect the prognosis due to the absence of eye care facilities and screening centers [14,16]. Addressing these issues is not only a matter of improving vision, but also a step towards promoting educational equity, economic development, and health equity since the vision impacts many areas of life. The objective of this paper is to assess eye health awareness and barriers for individuals from disadvantaged and low socioeconomic communities to inform future interventions that improve awareness and access to eye care.

## 2. Methods

This is a pilot study that employed a structured online questionnaire that was designed to assess community-based patients’ demographics, eye health awareness, and access to eye care. This questionnaire served as a quality improvement survey that aimed to provide a comprehensive assessment regarding the awareness of eye health and accessibility to eye health services for underserved populations in the community. This study was a secondary data analysis of the initial survey that was given to community patients. Adults aged 18 or older were eligible to complete the survey. Those who could not understand English were excluded. The main targeted demographic variables were socioeconomic status, age, and education. This was a voluntary, anonymous survey with no follow up of name, date of birth, address, or telephone number.

A literature search was also conducted to evaluate the various barriers to eye care and strategies to improve community health outcomes. Search terms such as eye health awareness, eye disease prevention, public health education, community and health partnerships, and public health interventions were utilized in databases such as PubMed, Scopus, and Google Scholar. The primary goal was to develop a better comprehension of the current situation in consideration of eye healthcare within these communities and identify potential stakeholders to inform future strategies that can aid in improving eye health awareness and service availability. The study was conducted according to the guidelines of the Declaration of Helsinki. This study was approved by the University of Rochester Institutional Review Board with an IRB approval number STUDY00010307.

## 3. Results

**Figure 1:**
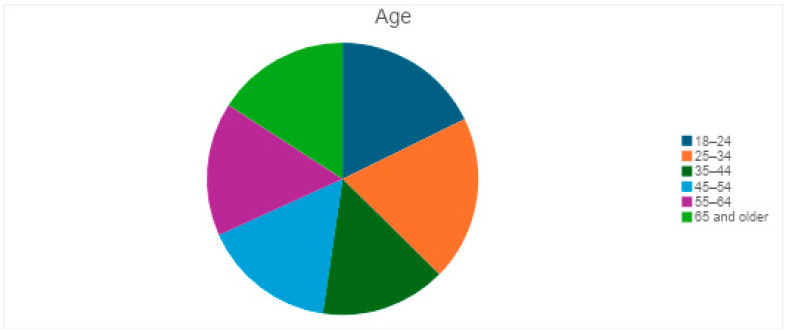
Participant age distribution.

**Figure 2:**
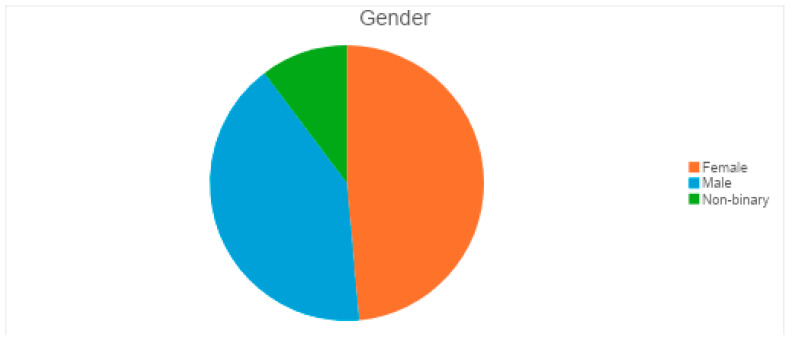
Participant gender.

There was a total of 107 respondents to the survey. The majority were aged 25–34 (19.6%), followed by 18–24 (17.8%), 35–44 (15.0%), 45–54 (15.9%), 55–64 (15.9%), and 65 and older (15.9%). This balanced distribution helps to analyze eye health awareness across different age groups (Figure 1). The gender distribution shows 52 females, 44 males, and 11 non-binary participants (Figure 2). The near-equal split between males and females ensures diverse perspectives on eye health issues. Including non-binary respondents allows for a comprehensive understanding of demographic differences in healthcare access.

**Figure 3:**
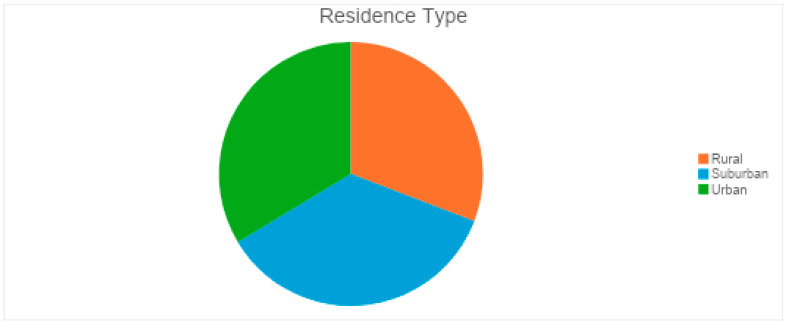
Residence type of respondents.

**Figure 4:**
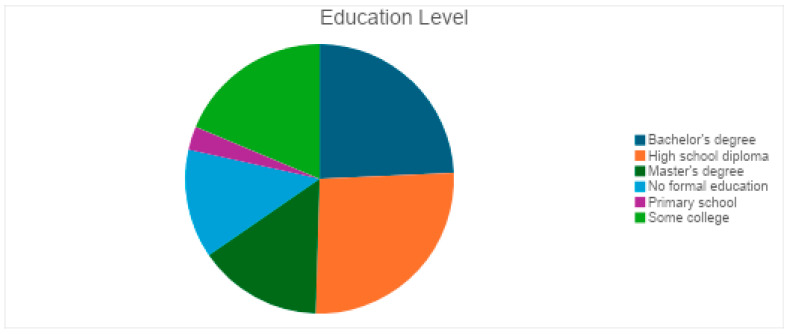
Education level of participants.

**Figure 5:**
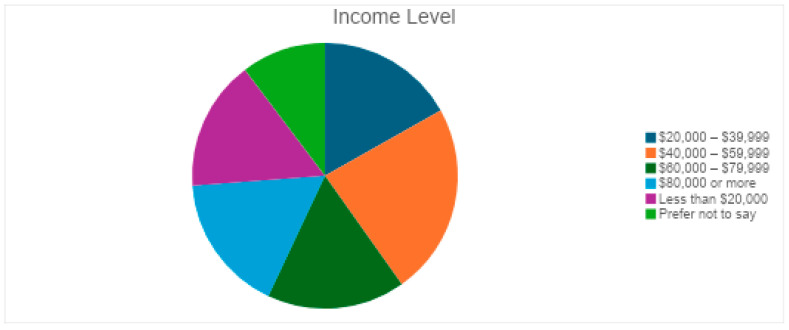
Income levels of participants.

The respondents had a balanced distribution of residence type, which helps assess geographic disparities in eye health accessibility, highlighting whether urban, suburban, or rural populations face greater challenges in seeking medical attention for vision-related issues (Figure 3). Participants had varying educational backgrounds: 26 held a bachelor’s degree, 28 had a high school diploma, 16 had a master’s degree, 20 attended some college, 14 had no formal education, and 3 had primary school education (Figure 4). The financial background of respondents varied, with 25 earning $40,000–$59,999, followed by 18 each in the $20,000–$39,999, $60,000–$79,999, and $80,000 or more ranges. Seventeen earned less than $20,000, and 11 preferred not to disclose their income (Figure 5).

### 3.1. Eye Health Awareness of Participants

**Figure 6:**
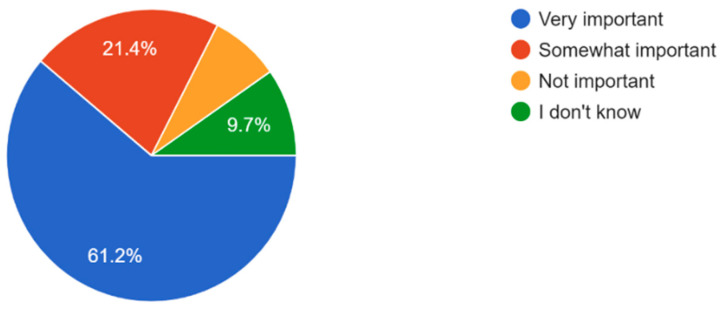
Respondents’ beliefs of importance of regular eye check-ups.

**Figure 7:**
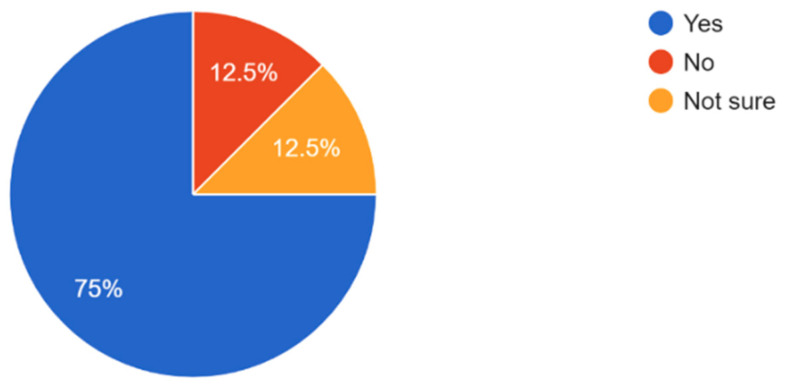
Do participants know that regular eye exams can help prevent serious eye conditions?

**Figure 8:**
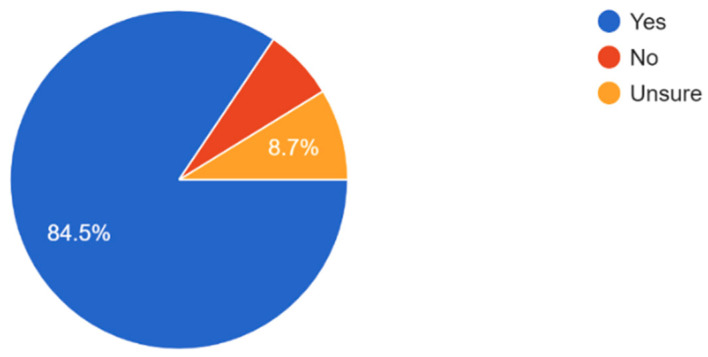
Respondents’ beliefs on whether eye health can affect overall quality of life.

**Figure 9:**
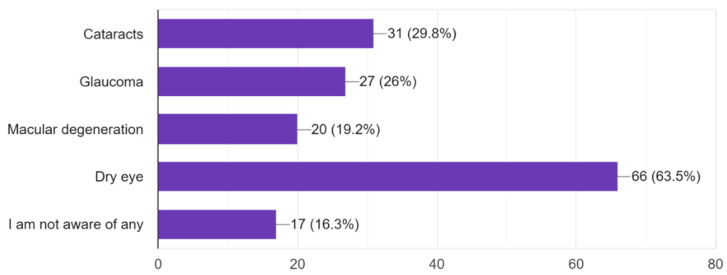
Eye conditions that are known amongst study participants.

**Figure 10:**
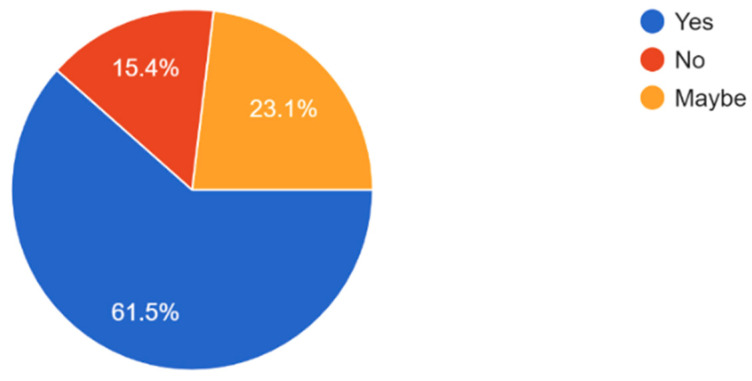
Proportion of participants interested in attending an eye health awareness program or workshop in their community.

The majority of participants understand the importance of regular eye exams with 61.2% of respondents believing that eye exams are very important and 7.7% believing that regular eye check-ups are not important (Figure 6). Most participants exhibited general awareness of the importance of eye exams in preventing serious eye conditions and how eye health affects overall quality of life (Figure 7 and Figure 8). Overall, dry eye (63.5%) was the most widely known eye condition amongst the participants, followed by cataracts (29.8%), glaucoma (26%), and macular degeneration (19.2%) (Figure 9). Moving forward, 61.5% of participants expressed interest in attending eye health awareness programs or workshops in their communities (Figure 10).

### 3.2. Barriers to Access Eye Care

**Figure 11:**
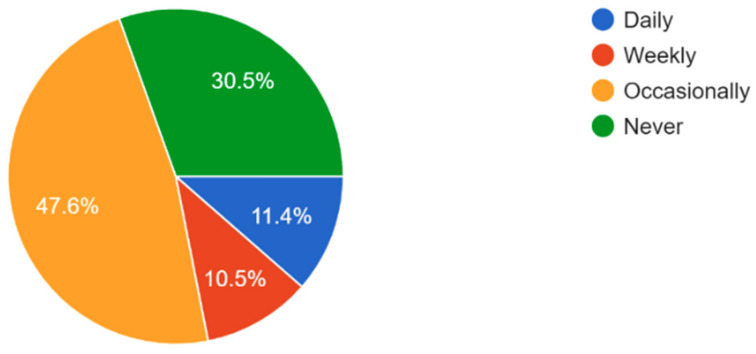
Frequency of vision problems experienced by survey respondents.

**Figure 12:**
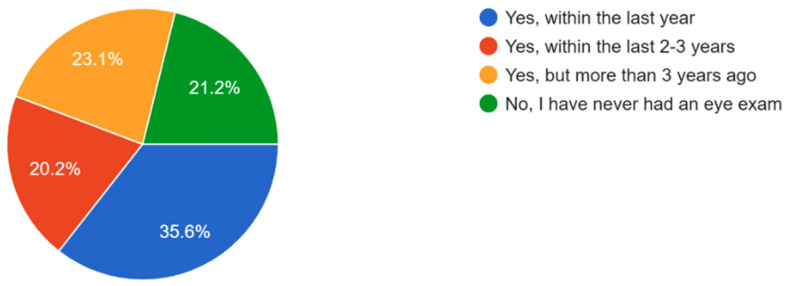
Participants who have had their eyes checked by a healthcare professional.

**Figure 13:**
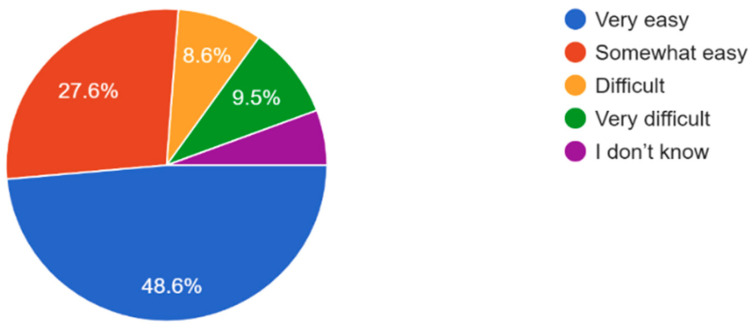
Ease of access to eye care services in the community.

**Figure 14:**
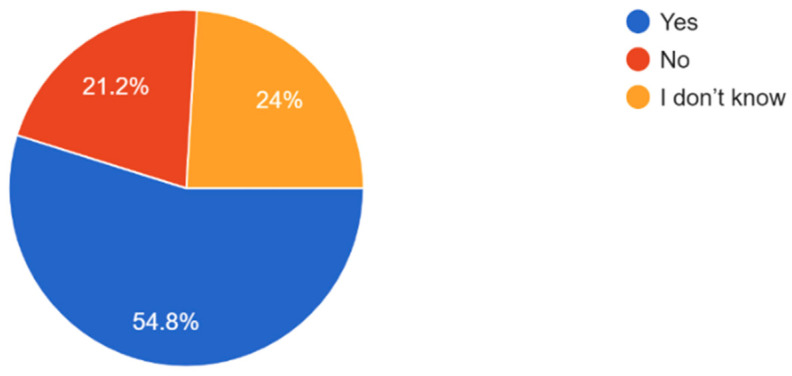
Availability of free or low-cost eye care services in the community.

**Figure 15:**
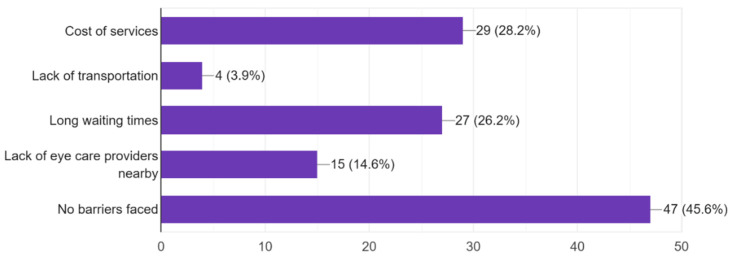
Barriers in access to eye care services faced by study participants.

The majority of participants experienced some form of vision problems such as blurred vision or eye strain, with 47.6% experiencing occasional visual problems (Figure 11). 35.6% of participants reported they had their eyes checked by a healthcare professional within the last year, while 21.2% reported never having an eye exam (Figure 12). Survey results highlighted that respondents mostly found access to eye care services in their community somewhat or very easy (Figure 13). In regard to the availability of low-cost eye care services in communities, 54.8% of participants reported having low-cost eye care while 21.2% reported not having low-cost eye care (Figure 14). Although 45.6% of participants reported not facing any barriers regarding access to eye care, the cost of services, long waiting times, and lack of nearby eye care providers were often cited as barriers from the remainder of the participants (Figure 15).

## 4. Discussion

One of the primary findings acquired from the survey is associated with the significant requirement for increases in awareness and access to eye healthcare within marginalized and underserved communities. Even though most of the respondents (78 participants) indicated that they had an extensive understanding of the importance of regular eye exams for preventing the development of significant eye conditions, many still indicated a lack of understanding and a lack of feasible access to eye healthcare services (Figure 7 and Figure 13). This finding is in alignment with the existing literature which highlights that having only awareness regarding the importance of maintaining eye healthcare is not adequate for improving eye health outcomes among the population without the availability of accessible services [17,18]. Previous studies have indicated that there is an extensive lack of accessible and affordable eye care services which continues to be one of the most critical barriers to maintaining positive outcomes among the marginalized population in terms of eye health [17,18]. Existing barriers include financial constraints, long waiting times, and a lack of availability of nearby eye care providers. All these barriers either prevent participants from receiving adequate care or deter them from wanting to receive care. With a significant shortage in eye providers such as ophthalmologists, the current workforce is not adequate to meet the demand of the growing population [19]. Hence, initiatives such as the expansion of residency programs need to take place in order to increase eye provider workforce. Additional large-scale campaigns and screening programs (telehealth or in-person) conducted by eye health professionals can also assist in increasing eye health access.

The survey indicated that even though the majority of individuals found varying degrees of easy access to eye care, 9 participants found it to be significantly difficult while 10 reported very difficult accessibility to eye care (Figure 13). Challenges such as long waiting times, geographical barriers, cost, and lack of eye professional availability induce a reduction in the timely care of eyes and often the reduction in the motivation of the individuals to acquire regular eye exams for maintaining appropriate eye health. These results highlight the necessity of developing more affordable, competent, and accessible eye care options within marginalized populations and communities such as the introduction of free or subsidized services and expansion of telehealth services and mobile clinics.

Similarly, the survey further indicated that although dry eye was the most acknowledged eye complaint, there seems to be little awareness of more severe conditions such as cataracts, glaucoma, and macular degeneration (Figure 9). It is not uncommon for people to lack knowledge about more severe, often painless, forms of eye diseases and instead seek attention when they notice more systemic or painful problems [20]. This indicates the potential for health educational campaigns and workshops on the commonly known diseases and the necessity of regular eye checkups that can minimize the worsening of vision impairment due to these diseases.

A reason that access to healthcare services is a problem in low-income communities is that individuals are unaware of the available services [21]. Hence, various community awareness programs can help increase the usage of services by raising knowledge about them in the communities. Although 54.8% of respondents had access to affordable and/or free eye care programs, 21.2% did not and another 24% appeared not to know or were in doubt (Figure 14). Other studies have found that in low-income communities, many individuals do not have access to information pertaining to resources for free or discounted medical care, resulting in underutilization of the care and subsequent worsened quality of life and outcomes [22,23]. Thus, there remains a need to utilize better approaches to alert socially excluded communities to the availability of these services.

Over half (64) of the participants declared an interest in attending eye health awareness programs or workshops suggesting a willingness to participate in local health initiatives (Figure 10). Community engagement programs and activities are precious resources for enhancing health education. Research has shown that culturally grounded initiatives emphasizing local community members are effective to increase knowledge and promote sustainable good practices regarding health [24]. The information obtained from the survey implies that it is crucial to involve religious, educational, and other community leaders to promote community health and well-being.

Several respondents (29) believe that cost is a barrier to eye care for them; others are disadvantaged because of the distance to eye care services or lack thereof (Figure 15). Such challenges are well depicted in the literature, where issues of transportation have long been identified as problematic, especially by patients in low-income or remote areas [25]. It is therefore useful to work with local health centers, relevant governmental and non-governmental health facilities, and other relevant NGOs to help address health access barriers. Increased accessibility options for service delivery include mobile clinics, telehealth, or affordable care options.

Paraprofessionals such as community health workers are people easily trusted within community settings and can educate individuals on eye health education and screening in addition to referring patients to the appropriate facilities. The WHO recognizes community health workers as a crucial workforce in expanding healthcare provisioning in the populations left behind [26]. Mobile eye clinics that entail going to remote areas in association with governmental and public health programs that offer affordable eye care could also be increased to address lack of access to healthcare. Increasing these early initiatives would effectively fill these knowledge and access deficits. Public health officials, healthcare stakeholders, and community leaders need to address these barriers through a multitude of strategies such as implementing educational campaigns, increasing availability of services, developing collaborations, and creating local health initiatives (Table 1). Each community might require different or multiple interventions pertaining to the specific needs. Nevertheless, it is crucial that future research continues to be conducted based on the health knowledge, literacy, and access of different populations and communities in order to ensure fair and equitable care.

### 4.1. Identifying Stakeholders

Many communities experience barriers to healthcare because of resource constraints, health literacy, and other population demographic factors, making it necessary to identify and involve key stakeholders for policy and practice change in eye health. Local and national public health departments are involved in carrying out eye health programs, coordination of awareness promotion, and scheduling of screenings, especially for vulnerable communities. To implement effective eye health interventions in the target communities of low and middle-income populations, stakeholders such as ophthalmologists, nurses, optometrists, and other eye care specialists are useful in guaranteeing that people in low-index areas receive appropriate medical attention, eye check-ups, and complete treatment (Table 2).

Local and national authorities are important determinants of the public health agenda for any given system [27]. They can allocate resources toward eye health campaigns, prescribe eye care services in their policies, and govern the industry. Their support is essential for the promotion of systematic changes for eye care; for example, lifting financial or organizational hindrances and distribution of resources on an equal basis. Religious as well as school authorities and other people who hold certain authorities within the community work to establish trust and can increase awareness of the importance of eye care, available treatment options, and eye care programs. They can enhance the level of participation of the public in screenings and awareness of various health conditions (Table 2).

Non-governmental organizations (NGOs) play a key role in addressing areas which have been given relatively low attention as they supplement the government’s efforts in terms of funding, material support, and lobbying [28]. They can also supply specialized services like mobile clinics or low-cost eye care services where the government might not fill the void. NGOs commonly assist marginalized groups and can make an enormous difference in the availability of resources to patients. Various eye health promotion strategies are implemented and supported by local and national public health organizations. These agencies can coordinate awareness creation, screening, and public health activities aimed at the uncovered populations. Consequently, they can engage as effective champions of eye health and assist with implementing eye health into general public health platforms. Referring to a rather wide and effective network of key stakeholders can increase collaboration and optimize the outcome of the work performed to improve the visual outcomes of the community (Table 2).

### 4.2. Building Coalitions and Partnerships

Making changes to eye health for those in low-resource settings requires partnerships with healthcare providers, policymakers, community leaders, and non-governmental organizations. Several strategies can be used to enhance collaborative partnerships. A look at the Chapman partnership to help homelessness in Miami and Homestead, Florida shows that partnership models are basic forms of collaboration, which can be utilized to assist those in need [29]. Such formal arrangements thus create opportunities for resource sharing, information and expertise, and relationships to coordinate towards agreed objectives. For example, partnerships such as EyeCare Partners can be created to address issues of accessibility and use of eye-related health services [30]. Thereafter, integration platforms enabled the declaration of responsibilities and accountabilities. Whenever the stakeholders know where they stand, what is expected of them, and the intended results, future plans and initiatives become more efficient through improved coordination and the utilization of resources (Table 3).

Goals and objectives are very important: their achievement being the driving force that makes stakeholders have a common purpose. For instance, bringing attention to the possible eradication of preventable blindness produces a shared vision that brings healthcare professionals, policymakers, and community leads together. This centralized approach allows for focus on the same tangible goals. Finally, viable case studies like the Global Health Commission on Global Eye Health are available to guide future initiatives [13]. All these endeavors demonstrate how international organizations, governments, and local caregiver partnerships bring about improvements in people’s health in a particular country. By adopting similar models for other regions that have not been served appropriately, people will be able to have improved eye health through community partnerships and initiatives.

### 4.3. Proposing Actions: Community Engagement Strategies

Engagement of communities in particular is important when it comes to enhancing both eye and overall health within the economically disadvantaged and marginalized populations. A more effective approach is educational interventions within communities [31]. These programs can be implemented in local schools, religious institutions, community centers, and online. Inviting highly respected people from the community like religious leaders, teachers, and other role models to spread awareness also forms part of the strategies used to escalate effectiveness and coverage. Studies show that members of the communities encourage people to adopt positive health behaviors, such as getting routine eye checkups and comprehending the danger of eye illnesses [24]. Earlier diagnosis of eye diseases is crucial to preserve sight and health, with many eye illnesses being underdiagnosed in disadvantaged populations.

Clinics and other healthcare facilities can proactively arrange workshops and mobile clinics to increase access to eye care providers for screenings, consultations, and treatments. Moreover, the use of radio, social networks, and local television can become effective in reaching more people for public health campaigns. Such campaigns can call for timely diagnosis of diseases like cataracts, glaucoma, and macular degeneration that few people consider or even know. In addition, peer-education programs can be implemented to teach the members of the community about the general health of the eyes, so that they can educate other members of the community, which makes it a sustainable model of education [32].

### 4.4. Recommendations for Collaboration with Local, State, and National Organizations

For these initiatives to work effectively, it is important to engage the support of local, state, and national organizations associated with eye health. Hospital personnel need to play a major role in mobilizing for eye screenings and for the actual treatment, especially where non-governmental organizations may provide funding and logistic support for such campaigns. Similarly, health and poverty-fighting non-governmental organizations like the Lions Club International have been instrumental in enhancing the provision of eye care services to the needy through the use of its mobile clinics and vision care [33]. Communities can obtain access to funds for implementing their projects and other critical resources by partnering with such organizations.

Feasible awareness programs can be conducted by the state and national departments of public health or CDC and WHO where they can provide awareness content and subsidies for eye care services [34,35]. Local programs can also provide funding to establish grants or policy advocacy for the creation of improved distribution systems for eye care. We strongly recommend that efforts should be pushed for such government-sponsored affordable or even free eye health services such as mobile eye care clinics to increase access to eye care for rural and underserved populations. These organizations can also be useful in mentoring the existing human resources in developing, often underserved, regions. To encourage the synergy of these groups, it is possible to form multi-sectoral partnerships [36]. These coalitions would act as partnerships through which healthcare providers, representatives of communities, and public health departments work towards providing care, increasing awareness, and policy formulation for eye health for the underserved communities.

### 4.5. Communication Strategies for Different Audiences and Sectors

Communication is central in public health, given that its principal pillar entails raising awareness or instilling knowledge and positive attitude/behavior change. Successful interpersonal communication can help to influence the promotion of healthy behaviors, promote routine screenings, and increase awareness about more severe, lesser-known conditions. Some of the critical audiences that public health workers should address regarding eye care are the general population, healthcare practitioners, and the government. The public needs general information about vision care, refractive errors, eye diseases, importance of eye exams, and availability of accessible options for the exams. Communications should be clear, easy to comprehend, and appeal to the target population (Table 4).

There is a need for leaders to facilitate diverse informative avenues that can make previously neglected populations more aware of their eye health. They can use simplified visually appealing materials which include but are not limited to infographics, facts and figures, and pamphlets. These materials should be devoted to informing the reader about major eye diseases such as cataracts, glaucoma, and macular degeneration, the main symptoms of diseases, and the need to visit an eye care provider regularly. Brochures and infographics should be provided at healthcare centers, community events, and other public areas so that anyone interested can easily access the information (Table 4) [37].

Community workshops are also recommended for community outreach since they can be held in various educational institutions, recreational, and community facilities such as schools, churches, and clinics [38]. They create a chance for healthcare professionals to engage people physically and respond to the questions or concerns people may have about their eyes. These workshops may provide an opportunity to consolidate concerns, which may be applicable to the community and its cultural values on issues to do with healthcare. Workshops can help make the information delivered more appropriate to the specific needs of the community. The use of local leaders in the dissemination of eye health messages is very important. Religious leaders, teachers, and social workers are people who can spread information about health initiatives in a culturally sensitive manner that fits the needs of the community. This makes it easier for people to listen and act based on words from trusted community leaders.

To reach a wider audience, public health educational campaigns through forms of radio, TV, social media, and local newspapers can enable access to a wider audience. Campaigns can be very useful in informing the public of the availability of cheap or even free eye care services, mobile eye care clinics, and nearby doctors that offer their services at low rates. In addition, making a campaign lively with illustrative stories and visuals which add value and makes it more personal, allowing the population to relate to stories and take positive steps to ensure they protect their eyes [39]. One can enrich the message by adding narrations and case histories, which can increase the chances of people going for an eye care check if the testimonies mirror some of the issues that the target group faces. These communication approaches help public health campaigns to raise awareness levels, encourage the population to take necessary activities, and respond positively to improve the eye health of the disadvantaged populations and minority groups.

Additionally, specific strategies can also be used to inform healthcare workers about current developments in eye health. The use of workshops and continuing education sessions enables the people who deal with eye care to improve their competence in the area by informing them on the current practices [40]. The topics that may be addressed under these workshops may include recent developments in cataract surgery, new techniques in imaging for diagnosis, and updated strategies and treatments related to dealing with chronic eye illnesses like glaucoma and diabetes retinopathy (Table 5).

Knowledge transfer of evidence-based practices entails disseminating current research information, clinical recommendations, or case studies with the view of passing the information to other stakeholders via newsletters, conferences, or online sources. Through the provision of guidelines and protocols that outline the best practice, healthcare professionals can utilize up-to-date evidence-based practice, improving the quality of the clinical decisions. Healthcare workers such as optometrists, ophthalmologists, and other related specialists stand to benefit from this strategy as well as organizing multidisciplinary sessions to collaborate with each other and stay informed of new developments within the field of eye care (Table 5). These collaborative sessions and organizations can also raise awareness of the health status of various communities and underrepresented populations to improve targeted interventions such as creating screening centers and providing resources to certain locations.

Additional strategies can be utilized in order to change legislation and promote funding for public health programs when communicating with policymakers. Carefully constructed policy briefs and presentations that are clear and credible remain useful in getting stakeholders to appreciate the need for addressing eye health complications in the target populations [41]. These documents present summarized, peer-reviewed data on the present conditions of eye health, inequalities in service provision, and the possible effect of reforms. Thus, by depicting these in formal meetings, the change in policies, funding, and resources required in eye care can be well understood (Table 6).

There are also advocacy and lobbying strategies that can be adopted as an effective tool in presenting public health issues. All advocacy approaches can entail providing such facts and figures in support of the cause, sharing real-life experiences of people affected by the problem, and/or involving relevant authorities or experts to encourage the need for positive change to improve eye health. In this way, such attempts can gather people’s support and attract the attention of decision-makers to invest in better services and pass enabling legislation (Table 6) [41].

In addition, such policy actions can take the form of dialogs and forums which make discussions more inclusive of the policymakers, healthcare providers, and other experts. They can be used to create common ground on how to approach the eye health sector, its difficulties, and its possibilities. Research is essential in persuading policymakers on the importance of putting resources into eye health programs. Information and facts like reports, cases, and research also can help persuade policymakers to assist in making positive changes in the overall health and resources for a population [42].

For the enhancement of initiatives in the reduction in blindness, communication is crucial, especially for disadvantaged communities. For the general population, there is awareness creation through the production of simple language pamphlets, organizing community health fairs, and enlisting the support of local leaders, among others. They assist in narrowing down the existing health literacy gap and foster the pursuit of good eye health among people. Additionally, healthcare professionals need to engage in continued professional development embracing evidence-based and improved practices as well as working collaboratively. Furthermore, policy decisions such as policy briefs and advocacy are crucial to be able to obtain the political support and appropriate funding for promoting eye health in the community.

### 4.6. Limitations

This study has several limitations including the small sample size due to its pilot nature, which limits the generalizability of the results. The questionnaire was not validated because it was created to assess the needs and awareness of the study population. This pilot survey and study will be used to inform larger-scale studies and initiatives to create a better way of reaching out to patients using implementation science to include more diverse groups in order to improve access and health outcomes. This study also did not collect any race/ethnicity data, which would be valuable to include in the future.

## 5. Conclusions

This study demonstrated that even though there is a good understanding of the importance of timely and adequate eye health, especially the underserved communities, many people still are unaware of the importance of eye health and lack access to adequate resources. As a result, disadvantaged communities often have worse outcomes regarding ocular health. Moving forward, local interventions such as public health workshops, mobile eye clinics, and telehealth are effective options to obtain an understanding of a patient’s situation. Afterward, if needed, the patient can be guided and referred for an in-person follow-up at a healthcare facility. Moreover, policymakers and stakeholders at all levels need to form respective policies, ensure funding of public health programs, and promote access to eye care services. In order to appropriately receive support and awareness of eye conditions, effective and informative communication strategies tailored to the audience are needed. By using implementation science with community-based research management, we can create a better way of reaching out patients to improve eye health awareness. It is only when all the gaps in eye health services have been improved through a collaborative approach and when the necessary resources have been committed and continuously mobilized that the goal of increasing access and availability of eye care to needy people is achieved. As medicine continues to evolve, it remains crucial to ensure all people have access to similar opportunities of care to meet their needs.

## Figures and Tables

**Table 1:** Recommendations for future interventions in communities to increase healthcare access.

Recommendation	Description
Increase public health campaigns to raise awareness of common and serious eye conditions.	Implement targeted campaigns to educate the public about common eye conditions like refractive problems, cataracts, glaucoma, macular degeneration, and even rare eye diseases as well as the importance of regular eye exams.
Expand access to affordable or free eye care services, including mobile clinics and public health programs.	Increase the availability of affordable eye care services through mobile clinics and public health initiatives to reach underserved populations.
Foster partnerships between local healthcare providers, non-profits, and public health organizations to address resource gaps	Develop collaborations between healthcare providers, non-profit organizations, and public health agencies to improve resource availability and coordination of eye care services.
Engage community leaders to promote eye health initiatives and create more educational opportunities for the public.	Involve local community leaders in promoting eye health, organizing awareness programs, and creating educational opportunities to raise awareness and improve eye health knowledge.

**Table 2:** Stakeholders in the health of communities.

Stakeholder	Role and Contribution
Local Healthcare Providers	Doctors, nurses, optometrists, and eye care specialists provide frontline medical attention, diagnostic services, and treatment options to the community.
Policymakers	Local and national policymakers influence public health priorities, allocate funding, and set regulations that improve access to eye care and advance policy reforms.
Community Leaders	Religious figures, educators, and local influencers hold community trust, spreading awareness and encouraging individuals to seek eye care services.
Non-governmental Organizations (NGOs)	NGOs working in health, education, and poverty alleviation provide resources, funding, and advocacy, and can offer specialized services to underserved communities.
Public Health Agencies	Local and national public health departments facilitate eye health programs, organize awareness campaigns, and implement screenings, particularly in underserved areas.

**Table 3:** Strategies to establish partnerships with community leaders and healthcare stakeholders.

Strategy	Description
Partnership Models	Establish formal partnerships where stakeholders pool resources, knowledge, and networks to achieve specific outcomes such as increasing access or improving awareness.
Collaboration Frameworks	Develop clear frameworks that define roles, responsibilities, and expectations, ensuring alignment of contributions towards common goals.
Shared Goals and Objectives	Align the vision of all stakeholders towards a unified objective, such as reducing preventable blindness, to create a sense of purpose and drive collaboration.

**Table 4:** Communication strategies for the public.

Strategy	Description	Purpose
Creating Easy-to-Understand Materials	Develop infographics, fact sheets, and simple brochures that explain common eye conditions, symptoms, and the importance of regular eye exams.	To increase awareness and make eye health information more accessible to a wider audience, including those with limited health literacy.
Community Workshops	Organize workshops and community events to educate individuals about eye health, available services, and preventive measures.	To engage the community directly and provide hands-on, interactive learning about eye care and its importance.
Public Health Campaigns	Launch media campaigns using radio, TV, social media, and local publications to reach a broader audience.	To raise awareness, build engagement, and inform the public about the risks of poor eye health and available services.
Storytelling and Testimonials	Share real-life stories and testimonials from community members or health professionals who have experienced eye health issues or improvement.	To make eye health issues more relatable and motivate individuals to take action, showing that eye health impacts real people.
Promoting Free or Low-Cost Services	Distribute information about available subsidized eye care programs, mobile clinics, or local healthcare centers offering affordable services.	To ensure that the underserved community knows about affordable or free eye care options, removing cost barriers to access.
Engaging Local Leaders	Involve respected community figures such as religious leaders, teachers, or social workers to deliver eye health messages in culturally appropriate ways.	To leverage trust and familiarity, ensuring that the message resonates within the community and fosters collective action for better eye health.

**Table 5:** Communication strategies for healthcare professionals.

Strategy	Description	Purpose	Expected Outcome
Professional Development	Organize training workshops and continuing education sessions focused on current eye care practices, new technologies, and diagnostic tools.	To enhance the knowledge and skills of healthcare providers in the diagnosis and management of eye conditions.	Improved competency in eye care practices and better patient outcomes.
Dissemination of Evidence-Based Practices	Share research findings, clinical guidelines, and case studies through newsletters, conferences, or online platforms that highlight evidence-based practices.	To ensure healthcare professionals are using the most current and effective methods in treating eye conditions.	Adoption of best practices and improved clinical decision-making.
Collaborative Learning Communities	Create communities of practice or peer learning groups where healthcare professionals can share experiences, discuss challenges, and learn from each other’s insights.	To foster knowledge exchange and mutual support among healthcare professionals.	Enhanced teamwork and shared knowledge to improve patient care.
Integration of Eye Health in Primary Care	Provide training for general healthcare providers to incorporate routine eye health screenings and discussions into general health assessments and appointments.	To increase early detection of eye conditions within primary care settings.	Early detection of eye diseases and referral to specialized care when necessary.
Digital Tools and Resources	Offer online platforms and mobile apps that provide easy access to the latest research, guidelines, and best practices in eye health for professionals.	To improve accessibility of eye care resources for healthcare professionals.	Streamlined access to up-to-date resources, facilitating quicker and better decision-making.
Multidisciplinary Training	Organize training programs that involve professionals from different sectors (e.g., optometrists, ophthalmologists, public health workers) to learn about eye health together.	To promote a holistic and integrated approach to eye care.	Enhanced coordination and collaboration among professionals for better patient care.

**Table 6:** Communication strategies for policymakers.

Strategy	Description	Purpose	Target Audience	Expected Outcome
Policy Briefs and Presentations	Create concise, evidence-based policy briefs and formal presentations summarizing the need for eye health initiatives, data on eye health disparities, and potential policy interventions.	To inform policymakers of the current state of eye health, the challenges in underserved communities, and the impact of policy reforms.	Policymakers (local, state, and national), health organizations	Increased awareness among policymakers of the need for comprehensive eye health policies and potential legislative actions.
Advocacy and Lobbying Strategies	Engage advocacy groups and lobbyists to influence policymakers by presenting compelling evidence, testimonials, and expert opinions on the importance of improving eye care access.	To mobilize stakeholders and build pressure for the implementation of pro-eye health policies, increased funding, and resource allocation.	Advocacy organizations, lobbyists, healthcare providers, local communities	Stronger advocacy for eye health policies, leading to the allocation of resources and funding for eye health programs.
Policy Dialogs and Forums	Organize and facilitate policy dialogs or forums that bring together experts, healthcare providers, and policymakers to discuss eye health priorities.	To foster discussions and build consensus on the necessary policy changes needed to improve eye health access and services.	Policymakers, health professionals, community leaders	Creation of a shared policy vision and collaborative efforts to improve eye health.
Evidence-Based Advocacy	Provide data-driven reports, research findings, and case studies on successful eye health initiatives and their outcomes.	To convince policymakers that investment in eye health programs will lead to measurable public health improvements and cost savings.	Policymakers, public health agencies, media	Policymakers are more likely to support eye health initiatives based on solid evidence.
Public Awareness Campaigns for Policymakers	Use public awareness campaigns to put pressure on policymakers by demonstrating public support for eye health reforms.	To create a public mandate for policymakers to prioritize eye health in policy agendas.	The general public, media, policymakers	Increased public demand for better eye health policies and greater political will to implement reforms.

## Data Availability

The original contributions presented in this study are included in the article. Further inquiries can be directed to the corresponding author.
